# The negative self-perceived health of migrants with precarious status in Montreal, Canada: A cross-sectional study

**DOI:** 10.1371/journal.pone.0231327

**Published:** 2020-04-09

**Authors:** Patrick Cloos, Elhadji Malick Ndao, Josephine Aho, Magalie Benoît, Amandine Fillol, Maria Munoz-Bertrand, Marie-Jo Ouimet, Jill Hanley, Valéry Ridde

**Affiliations:** 1 School of Social Work, University of Montreal, Montreal, Quebec, Canada; 2 School of Public Health, University of Montreal, Montreal, Quebec, Canada; 3 Centre de Recherche en Santé Publique (CRESP), Montreal, Quebec, Canada; 4 Department of Psychology, University of Montreal, Montreal, Quebec, Canada; 5 CEPED, Institute for Research on Sustainable Development, IRD-Université de Paris, ERL INSERM SAGESUD, Paris, France; 6 Department of Family Medicine and Emergency, Faculty of Medicine, University of Montreal, Montreal, Quebec, Canada; 7 School of Social Work, McGill University, Montreal, Quebec, Canada; 8 Fellow de l’Institut Français des Migrations, Paris, France; Graduate School of Public Health and Health Policy, City University of New York, UNITED STATES

## Abstract

**Background:**

Knowledge about the health impacts of the absence of health insurance for migrants with precarious status (MPS) in Canada is scarce. MPS refer to immigrants with authorized but temporary legal status (i.e. temporary foreign workers, visitors, international students) and/or unauthorized status (out of legal status, i.e. undocumented). This is the first large empirical study that examines the social determinants of self-perceived health of MPS who are uninsured and residing in Montreal.

**Methods and findings:**

Between June 2016 and September 2017, we performed a cross-sectional survey of uninsured migrants in Montreal, Quebec. Migrants without health insurance (18+) were sampled through venue-based recruitment, snowball strategy and media announcements. A questionnaire focusing on sociodemographic, socioeconomic and psychosocial characteristics, social determinants, health needs and access to health care, and health self-perception was administered to 806 individuals: 54.1% were recruited in urban spaces and 45.9% in a health clinic. 53.9% were categorized as having temporary legal status in Canada and 46% were without authorized status. Regions of birth were: Asia (5.2%), Caribbean (13.8%), Europe (7.3%), Latin America (35.8%), Middle East (21%), Sub-Saharan Africa (15.8%) and the United States (1.1%). The median age was 37 years (range:18–87). The proportion of respondents reporting negative (bad/fair) self-perception of health was 44.8%: 36.1% among migrants with authorized legal status and 54.4% among those with unauthorized status (statistically significant difference; *p*<0.001). Factors associated with negative self-perceived health were assessed using logistic regression. Those who were more likely to perceive their health as negative were those: with no diploma/primary/secondary education (age-adjusted odds ratio [AOR]: 2.49 [95% CI 1.53–4.07, *p*<0.001] or with a college diploma (AOR: 2.41 [95% CI 1.38–4.20, *p* = 0.002); whose family income met their needs not at all/a little (AOR: 6.22 [95% CI 1.62–23.85], *p* = 0.008) or met their needs fairly (AOR: 4.70 [95% CI 1.21–18.27], *p* = 0.025); with no one whom they could ask for money (AOR: 1.60 [95% CI 1.05–2.46], *p* = 0.03); with perception of racism (AOR: 1.58 [95% CI 1.01–2.48], *p* = 0.045); with a feeling of psychological distress (AOR: 2.17 [95% CI 1.36–3.45], *p* = 0.001); with unmet health care needs (AOR: 3.45 [95% CI 2.05–5.82], *p*<0.001); or with a health issue in the past 12 months (AOR: 3.44 [95% CI 1.79–6.61], *p*<0.001). Some variables that are associated with negative self-perceived health varied according to gender: region of birth, lower formal education, having a family income that does not meet needs perfectly /very well, insalubrious housing, not knowing someone who could be asked for money, and having ever received a medical diagnosis.

**Conclusions:**

In our study, almost half of immigrants without health insurance perceived their health as negative, much higher than reports of negative self-perceived health in previous Canadian studies (8.5% among recent immigrants, 19.8% among long-term immigrants, and 10.6% among Canadian-born). Our study also suggests a high rate of unmet health care needs among migrants with precarious status, a situation that is correlated with poor self-perceived health. There is a need to put social policies in place to secure access to resources, health care and social services for all migrants, with or without authorized status.

## Introduction

This paper contributes to the domain of migration and health, viewed as a global public health priority [1–3] that requires effective intersectoral policies [4] through coordinated action on the social determinants of health [5, 6]. The legal immigration classification in place in a country contributes to shaping the social position of migrants in their new society of residence by assigning authorized migration statuses (permanent or temporary) associated with differential levels of rights and social protection [7–9]. The legal classification of migration (and the exclusion of the unauthorized) shapes health and produces health disparities through differential access to resources [10,11], together with other forms of social hierarchy like gender, national origin or ethnicity and social class, which have the power to exacerbate the challenges faced by immigrants [7]. Migration status is seen as a social determinant of health in its own right [12–14]. It determines eligibility for public health insurance, which may vary from one period to another depending on the migration process and procedures [12]. An absence of health insurance [9,15,16], plus language barriers [17], are among the main reasons for immigrants not accessing medical services despite needs.

Since 1960 and the postcolonial era, more countries are contributing to the global economy and human mobility, making people’s international movements increasingly diverse and non‐European, with a growing attractiveness of wealthy destinations in Asia, Europe and North‐America [18,19]. In 2017, there were an estimated 258 million international migrants, around 3.4 per cent of the global population, an increase from 2.8% in 2000 [20]. States seek to control international migrations through various policies and regulations [21], and countries like Canada select migrants based on national origin, occupation and wealth [19]. Since the late 1990’s, the dominant Western discourse about human migration has been conflated with security issues, representing some migrants as potential threats to security and undesirable, whose control necessitates more drastic migration laws [22,23]. The vulnerability of migrants has increased and the erosion of their rights has been noted in many receiving countries, for example with the use of retention centers in France [24]. Migrants’ legal status and general wellbeing are the most precarious within the context of irregular migration, including unauthorized entry or stay within a country [18].

In 2011, the migrant population was 21% of the total Canadian resident population [3], a proportion that is projected to increase in the next decade. Canadian immigrants are predominantly born in Asia (44,8%), followed by Europe (31%), Central, South America and The Caribbean (11,7%) and Africa (7,2%). Montreal, Toronto and Vancouver are the primary areas of residence for immigrants in Canada. For the period 2013–2017, the province of Quebec accepted about 256,000 permanent residents, which represents a yearly mean of approximately 51,000 new immigrants [25]. Most of Quebec’s immigrants reside in Montreal, where they represent approximately 23% of the city’s population. In the near future, the migrant population of Montreal will be increasingly diverse, with the main places of birth being located in Africa, Asia and The Americas [26]. International migration is not only characterized by diversity of both geographical origin [2] and migration categories (labor, student, family, permanent and temporary) [19], but also by social inequities and poverty. In Canada, non-European born immigrants are generally more affected by chronic low income [27,28]. There is a universal and public health-care system in Canada, however some categories of immigrants (very recent newcomers, rejected refugee claimants and those whose work permit or visa is expired) do not have public insurance [5].

The association between immigration and health is not always clear and straightforward [29], and studies show contrasting trends [30]. Migrants represent a heterogeneous category whose health needs are largely unknown due to a lack of quality data, especially for certain subcategories like those who are undocumented [6]. To the best of our knowledge, there is an absence in the literature of a comprehensive model that takes into account interrelated factors—structural, intermediary and more individual—that is specific to immigrant health [11]. It was already suggested that recent immigrants to Canada, who are usually highly educated [11], have on average comparable or sometimes better health outcomes than the native population [30]. Some authors describe a *healthy immigrant effect* in wealthy countries such as Australia, Canada, Europe and USA: recent immigrants have a better physical and mental health status than national born citizens [17, 31–33]. However, these health trends seem to deteriorate with years spent in the host country and over time migrant health statuses become closer to the native-born population, as has been observed in Canada and elsewhere [2,3,31]. Racism [10,34], poor living and working conditions [35,36], stresses of integration [17,37], unemployment, lack of social networks and lack of access to services [11] or health care [38], and more globally, deprivation [38], are all factors of vulnerability associated with immigration and reasons for health to decline among recent immigrants. In general, new immigrants experience poor socioeconomic conditions, which may lead to a deteriorating health status, and even after improvement of social conditions, their health status declines to become similar to the Canadian-born population [3] (p.108). Gender might affect migrant health in interaction with SES, country of origin and age; lower paid and unsatisfactory jobs, children care, little social support, duration of residency in the host country, and chronic stress can also be a source of poorer health among women and subsequent gender inequalities among migrants [39].

Other studies suggest a *health paradox* associated with immigration in the US and Europe: some migrant ethnic minorities have good health outcomes despite low socioeconomic status [40,41]. A study on perinatal mortality in Belgium indicates a relative protective role of immigration, although this protection seems limited to women with low socioeconomic status (SES), and not those with higher SES [42]. This *health paradox* is not always observed as authors indicate an excess of perinatal mortality among non-European migrants women and this, independently of SES, at least for some nationalities [30]. Health benefits associated with immigration are not generalizable [37] but vary according to nationality [30,41] or places of birth [31,42]; racialized minority status [2,43]; SES [3,42,44]; gender and age [34,37,44]; duration of residency in the host country [3,44]; and migration status [10,15] or uninsured status [37]. In fact, migration status represents a risk for health [4, 7, 12], especially for those with precarious status [9] and *a fortiori* for those without authorized status (undocumented) [44], as they face multiple and additional deleterious challenges, some of which representing barriers for accessing health care [9].

In Canada, migrants can have two different levels of legal authorization: permanent status (economic, family and humanitarian categories of immigrants) or temporary status (ex. foreign workers, asylum seekers or international students) [25]. The concept of migrants with precarious status (MPS) refers to both people whose legal authorization to remain in the country is temporary and/or dependent on a third party (i.e. asylum seekers, temporary foreign workers, family members awaiting sponsorship, visitors, international students, live-in caregivers, victims of human trafficking) and people whose presence in the country is unauthorized (i.e. the undocumented) [9] (p.331). Precarious legal status limits or prevents access to state entitlements [45], and creates a situation of insecurity that can have long-term effects [46]. In 2017, in Quebec, there were approximately 107,000 people with precarious status related to work or study [47], while about 28,000 asylum claims were received in the province [25]. Undocumented migrants are individuals who most often entered the country through official channels but whose visa expired, who received a negative response for their immigration procedure or could not renew their visa but stayed in the country [48]; in contrast to the United States or Europe, only a small proportion of undocumented migrants in Canada are estimated to have entered the country unlawfully. Estimates of migrants without authorized status in Canada vary between 20,000 [44] and 500,000 [9]. In Quebec, all permanent residents as well as some temporary migrants are entitled to public health insurance through the *Régie de l’Assurance Maladie du Québec* (RAMQ). Asylum seekers are not eligible for RAMQ but may access equivalent services through the Interim Federal Health Program (IFHP). However, many migrants with valid temporary status, as well as those without authorized status, are excluded from any public health coverage [16,49]. In addition, there are many loopholes through which otherwise eligible permanent and temporary migrants may lack access to a public health insurance [9]. A few studies have documented the poor access to perinatal care [50,51] and perinatal outcomes [52] of uninsured pregnant women in Canada. However, there remains a dearth of information about the health impact of the absence of health insurance for migrants with precarious status in Canada, despite the fact that it seems their number is rising [21]. Furthermore, migrants with precarious status often express a fear of research teams and are consequently underrepresented in population-based research [53]. Considering this, the objective of this article is to examine the association between precarious migration status and self-perceived health in Montreal. More specifically, it aims to identify the social elements that can help to understand the potential deleterious correlates involved in the precariousness of migration status. This information could orient health programs and policies. We hypothesize that self-rated health among immigrants varies across status of migration, region of birth, gender and SES.

## Materials and methods

### Study design and participants

Between June 2016 and September 2017, we performed a cross-sectional survey of uninsured migrants in Montreal, Quebec. Inclusion criteria included being born outside of Canada and reporting not having health insurance (whether RAMQ, the IFHP or private insurance), being aged 18 and over, residing or intending to reside in the province of Quebec for more than 6 months and/or intending to obtain permanent residence. Individuals who were unaware that they were eligible for the IFHP or who had benefitted from it in the past but had not been able to extend or to renew it were also included. Exclusion criteria included: benefiting from a private insurance that covered all types of primary care with similar coverage as public insurance; being a Canadian citizen or a permanent resident; and being unable to communicate in one of the six languages of the study. Eligibility was assessed through an informal screening questionnaire that asked about trajectory of migration and medical insurance. Trained multicultural and multilingual research assistants administered the questionnaire through 30- to 90-minute face-to-face interviews using a tablet (OdK Collect software). There were two distinct recruitment processes. The first was a venue-based community sampling methodology, snowball sampling and an active campaign in social media, communities, and the local press were undertaken, as described elsewhere [53]. A formative assessment took place prior to the study [54]. Key informants from community organizations and from academia participated in 5 brain-storming sessions to identify neighborhoods and places where the study population was known—or likely—to gather. A list of these places was established and regularly updated in consultation with community organizations and other key informants. The listing included places of worship, community organizations, parks, stores, food banks, transit hubs, and community events. Prior to fieldwork and throughout the study, an awareness and community engagement campaign was conducted via social media, local newspapers and radio. All participants were given an information sheet and were encouraged to communicate the information to potential eligible participants. The second type of recruitment took place through the NGO Doctors of the World (DoW)’s health clinic in Montreal that caters to the uninsured migrant population. People attending the clinic and who had undergone initial screening by the NGO and confirmed not to have health insurance were approached and invited to participate in the study.

### Consent and research ethics

This research was approved by the Research Ethics Board of the University of Montreal (15.154). Information about the research was given orally and in writing to individuals prior to the interview. Written consent was obtained before the interview. If participants preferred not to give their name and signature, verbal consent was obtained. In this case, the interviewer had to sign a document stating that informed consent was obtained, questions were answered, and individual’s rights respected, as required by the Ethics Board of the University of Montreal. All individuals were free to answer questions or to withdraw at any time. Respondents obtained $20 compensation for their participation.

### Measurements

The questionnaire was developed using the Trajectory model to understand health disparities in immigrants/refugees [55]. Questions and scales used and validated in migrant studies or in studies of the general population were included when possible. The questionnaire included variables on: sociodemographic characteristics (age, gender, education, country of birth, number of years in Quebec, family situation); migration status (categories of authorized migration status in Canada or not); knowledge of English or French; socioeconomic status (occupation, income, formal education); material and psychosocial resources (food security, accommodation, social support) and barriers (fears, discrimination); a 21-item scale on post-migration stress [56]; health (psychological distress measured by the Kessler 6 scale, self-perceived health, diagnosed medical conditions, health issues in the last year); and health care access (unmet health care needs; use of health services and barriers).The questionnaire was pre-tested by phone with 5 participants per language (French, English, Spanish, Arabic, Mandarin and Haitian Creole) (N = 25). We recruited participants for the pre-test by leaving flyers in community organisations, including Doctors of the World (four organisations in total). Following the administration of the questionnaire, participants were invited to a focus group to discuss questions that they perceived as not clearly formulated or that were uncomfortable for them to answer. We held six focus groups, with five participants each. Our main dependent variable for this article’s analysis, self-perceived health, was measured by “In general, would you say that your heath is [bad, fair, good, very good, excellent]”. Self-perceived health is a valid and reliable way to assess individual’s global health (physical and mental health) and well-being [57].

### Statistical analysis

Descriptive analysis of sociodemographic, socioeconomic and psychosocial characteristics was performed on both sub-samples (health clinic or population-based participants) and on the whole sample, using means, standard deviation and proportions. Differences between the sub-samples were not statistically significant for the main dependent and independent variables thus, results of the sample as a whole (or stratified by migration status) are presented here. We assessed variables associated with the main dependent variable (self-perceived health). We dichotomize our main dependent variable into two categories: bad/fair (negative self-perceived health status) vs good/ very good/ excellent (positive self-perceived health status) [31,58]. Guided by the Social Determinants of Health conceptual framework of The World Health Organization [59], the following independent variables were assessed as potential correlates of negative self-perceived health status: structural determinants that relates to social position (socioeconomic status, migration status, gender, region of birth); and intermediary determinants related to working conditions (work contract, occupational injury), material circumstances (food security, insalubrious housing (measured as at least one of the following situation: presence of cockroaches, bedbugs, mice, rats, smell or spots of mold, water infiltration, flooding or water damage, or other situation), psychosocial factors (social support, discriminations, mental distress, stressors and fear related to migration status), health issues (in the past 12 months) and access to the health system (unmet health care needs, number of years without medical insurance). Mental distress was assessed using the Kessler Psychological distress scale, dichotomizing the score using a threshold of no serious mental distress (0–12) vs serious mental distress (13–24), as described in Kessler et al. [60]. Univariate analyses were performed using Pearson and McNemar chi-square tests for categorial variables and Student t and Wilcoxon-Mann-Whitney tests for continuous variables. Variables associated with the dependent variable in the univariate analysis at a P<0.2 were included in a multivariate logistic regression. Odds ratios, their 95% confidence intervals (CI) and their associated *p* values are presented. Age was included in the model as a potential confounder. A multivariate logistic regression stratified by gender was also performed Independent variables of interest were the same as those described above, with a caveat of not having a statistically significant difference in distribution of independent variables of interest among males compared to their distribution among females. Missing values were rare and did not exceed 5% for most variables except 3 variables for which missing values reached a maximum of 12%. They were assumed to be missing at random and excluded from the analysis. The model was run on respondents with complete observations for all variables. Goodness of fit was assessed using Hosmer and Lemeshow test and the model's chi square. Adjusted OR and their 95% CI are presented. A P<0.05 was considered statistically significant.

## Results

The interviewers met with 20043 individuals in Montreal to talk about the research project. Discussion about eligibility to participate in the research project was undertaken with 1659 individuals. Overall, 806 participants were recruited: 436 (54.1%) in urban spaces and 370 (45.9%) at the clinic. Based on their declaration, 421 (53.9%) were categorized as having an authorized migration status (with a valid student, work or visitor visa) and 360 (46%) without authorization (absence of a valid visa).

### Sociodemographic, socioeconomical and psychosocial characteristics

“Table 1” summarizes the descriptive comparison between the two groups: with authorized migration status (visitors, temporary workers, international students and dependents) and without authorized migration status (undocumented).

**Table 1:** Sociodemographic, socioeconomic and psychosocial characteristics of the sample by migration status of migrants without medical insurance in Montreal, 2016–2017 (n = 806).

Variables	Migratory status n (%)		Total N (%)
	Authorized migration status N (%)	Unauthorized migration status N (%)	
SOCIODEMOGRAPHIC CHARACTERISTICS (Question)			
Age (in years) (median, range)	35, 18–84	39, 18–87	37, 18–87
< 25	34 (8.1)	22 (6.1)	56 (7.2)
25–34	175 (41.6)	113 (31.4)	288 (36.9)
35–44	86 (20.4)	100 (27.8)	186 (23.8)
45–54	38 (9.0)	67 (18.6)	105 (13.4)
55–64	47 (11.2)	40 (11.1)	87 (11.1)
65+	41 (9.7)	18 (5.0)	59 (7.6)
Gender			
Female	295 (70.1)	200 (56.0)	495 (63.6)
Male	126 (29.9)	157 (44.0)	283 (36.4)
Marital status			
Married	258 (61.3)	124 (34.5)	382 (49.0)
Common law	27 (6.4)	31 (8.6)	58 (7.4)
Widow	26 (6.2)	11 (3.1)	37 (4.7)
Separated/Divorced	20 (4.7)	60 (16.7)	80 (10.3)
Single	90 (21.4)	133 (37.0)	223 (28.6)
Living with children <18 at home			
No	307 (72.9)	272 (75.6)	579 (74.1)
Yes	114 (27.1)	88 (24.4)	202 (25.9)
Region of birth			
Sub-Saharan Africa	65 (15.5)	58 (16.5)	123 (16.0)
Latin America	147 (35.2)	128 (36.4)	275 (35.8)
Asia	23 (5.5)	15 (4.3)	38 (4.9)
Carïbbean	33 (7.9)	74 (21.1)	107 (13.9)
United States	5 (1.2)	3 (0.9)	8 (1.0)
Europe	37 (8.9)	19 (5.4)	56 (7.3)
Middle East	108 (25.8)	54 (15.4)	162 (21.1)
Duration of stay in Quebec			
5 years and over	16 (3.8)	162 (45.8)	178 (23.1)
Less than 5 years	401 (96.2)	192 (54.2)	593 (76.9)
SOCIOECONOMIC CHARACTERISTICS			
Education			
Primary school	27 (6.5)	38 (10.9)	65 (8.5)
Secondary school	78 (18.7)	100 (28.7)	178 (23.2)
Post-secondary non-university Diploma	67 (16.1)	85 (24.3)	152 (19.8)
University diploma	233 (55.9)	118 (33.8)	351 (45.8)
No diploma	12 (2.9)	8 (2.3)	20 (2.6)
Main occupation			
Work with salary	93 (22.7)	178 (50.3)	271 (35.5)
Volunteer (work without salary)	14 (3.4)	16 (4.5)	30 (3.9)
Student	45 (11.0)	11 (3.1)	56 (7.3)
Children caregiver	107 (26.2)	52 (14.7)	159 (20.8)
Other (looking for work, pension, etc.)	150 (36.7)	97 (27.4)	247 (32.4)
Type of work contract			
Permanent	21 (5.0)	53 (14.7)	74 (9.5)
Temporary	24 (5.7)	24 (6.7)	48 (6.1)
On call | Self-employed person	48 (11.4)	99 (27.5)	147 (18.8)
Not applicable (declared not working)	328 (77.9)	184 (51.1)	512 (65.6)
Family income meets needs			
No/a little	251 (64.2)	237 (71.4)	488 (67.5)
Fairly	119 (30.4)	82 (24.7)	201 (27.8)
Very well/perfectly	21 (5.4)	13 (3.9)	34 (4.7)
Insalubrious housing			
No	297 (71,6)	212 (59,4)	509 (65,9)
Yes	118 (28,4)	145 (40,6)	263 (34,1)
Food insecurity			
Never	279 (73.4)	183 (56.0)	462 (65.3)
Often | Sometimes	101 (26.6)	144 (44.0)	245 (34.7)
PSYCHOSOCIAL CHARACTERISTICS			
Discrimination at work			
No	344 (82.9)	244 (68.5)	588 (76.3)
Yes	71 (17.1)	112 (31.5)	183 (23.7)
Discrimination when accessing healthcare			
No	136 (32.7)	122 (34.6)	258 (33.6)
Yes	280 (67.3)	231 (65.4)	511 (66.4)
Discrimination based on origin (Racism)			
No	222 (53.2)	150 (42.1)	372 (48.1)
Yes	195 (46.8)	206 (57.9)	401 (51.9)
Discrimination because of migration status			
No	135 (32.5)	66 (18.4)	201 (26.0)
Yes	280 (66.7)	292 (81.6)	572 (74.0)
Fear/stress to obtain health care			
No	110 (26.4)	109 (30.7)	219 (28.4)
Yes	307 (73.6)	246 (69.3)	553 (71.6)
Fear/stress of meeting a police agent			
Non	348 (83.3)	182 (50.8)	530 (68.3)
Oui	70 (16.7)	176 (49.2)	246 (31.7)
Fear/stress related to immigration services			
No	Not applicable	55 (15.3)	55 (7.0)
Yes	Not applicable	239 (66.4)	239 (30.6)
Fear/stress when accessing public services			
No	Not applicable	100 (35.0)	100 (35.0)
Yes	Not applicable	186 (65.0)	186 (65.0)
Stress because of limited contacts with family and friends			
No	Not applicable	113 (39.9)	113 (39.9)
Yes	Not applicable	170 (60.1)	170 (60.1)
Having someone to talk to			
No	71 (16.9)	90 (25.4)	161 (20.7)
Yes	350 (83.1)	265 (74.6)	615 (79.3)
Having someone to who could be asked for money			
No	151 (36.7)	160 (46.4)	311 (41.1)
Yes	260 (63.3)	185 (53.6)	445 (58.9)

In terms of psychosocial characteristics, most participants (76.3%) did not feel discriminated against at work (especially if they had an authorized migration status) but felt discriminated against when accessing healthcare (66.4%), or felt discriminated against because of their origin (racism) (51.9%) and migration status (74.0%). Most participants, from both groups, expressed fear of obtaining health care (71.6%). Migrants without authorized status also reported fear of authorities such as meeting police (49.2%) or interacting with immigration services (66.3%) or public services in general (65.0%). They also reported stress related to feeling the need to limited contacts with family and friends (60.1%) although they had someone to talk to (74.6%) or to ask money to (53.6%).

### Health characteristics

As indicated in “Table 2”, 348 (44.6%) of all migrants perceived their heath as negative: 152 (36.1%) among migrants with authorized status and 196 (54.4%) without authorized status (statistically significant difference; *p*<0.001). Mental distress was reported among 192 (26,3%) of all individuals: 83 (20,9%) and 109 (32,6%) among migrants with and without authorized status respectively. Most participants (83.5%) reported a health problem within the previous 12 months. Half of the participants without authorized status reported having been without medical insurance for over 3 years. Unmet health care needs were declared among 527 (68,9%) of all respondents: 281 (67,4%) and 246 (70,7%) among migrants with and without authorized status respectively.

**Table 2:** Health characteristics of migrants without medical insurance in Montreal, 2016–2017 (n = 806).

Variables	Migration status n (%)		Total N (%)
	With authorized status	Without authorized status	
Health perception			
Good/ Very good/ Excellent	269 (63.9)	164 (45.6)	433 (55.4)
Bad / Fair	152 (36.1)	196 (54.4)	348 (44.6)
Kessler mental distress scale			
No (0–12)	314 (79.1)	225 (67.4)	539 (73.7)
Yes (13–24)	83 (20.9)	109 (32.6)	192 (26.3)
Previous diagnosis of a health problem (ever)			
No	271 (64.4)	224 (62.4)	495 (63.5)
Yes	150 (35.6)	135 (37.6)	285 (36.5)
Had a health problem in the past 12 months			
No	86 (20.4)	43 (11.9)	129 (16.5)
Yes	335 (79.6)	317 (88.1)	652 (83.5)
Occupational injury			
No	133 (94.3)	205 (82,7)	338 (86.9)
Yes	8 (5.7)	43 (17.3)	51 (13.1)
Number of years without health insurance	Mean:0.9	Mean:4.0	Mean:2.3
	Median:0.0	Median:3.0	Median:1
	Range : 0–13	Range : 0–27	Range:0–27
Unmet health needs since without health insurance			
No	136 (32.6)	102 (29.3)	238 (31.1)
Yes	281 (67.4)	246 (70.7)	527 (68.9)

### Correlates of negative self-perceived health status

“Table 3” presents the results of our multivariate analysis. In this analysis adjusted for age, the odds (OR) of perceiving one’s health status as negative were significantly higher among those not having a diploma or having only completed primary or secondary school (OR: 2.49, 95% CI:1.53–4.07) and having a college diploma (OR: 2.41, 95% CI:1.38–4.20) compared to those with a university degree. On the socioeconomic level, the odds of perceiving one’s health status as negative were also significantly higher among those who reported their family income only met their needs a little or not at all (OR: 6.22; 95% CI:1.61–23.85) or met them fairly (OR:4.70, 95% CI:1.12–18.27) compared to those who stated that their family income met their needs very well or perfectly. Similarly, not having someone who could be asked for money in case of need was significantly associated with a negative perception of health status (OR:1.60, 95% CI:1.05–2.46). Those who reported having experienced racism were also at higher odds of negative perception of health (OR: 1.58, 95% CI:1.01–2.48). Finally, mental distress (OR: 2.17, 95% CI:1.36–3.45), having had a health problem in the past 12 months (OR: 3.44, 95% CI:1.79–6.61) and unmet health needs in the past 12 months (defined as not having been able to access health care when needed) were significantly associated with a negative self-perceived health status (OR: 3.45, 95% CI:2.05–5.82). There was no statistical association between no authorized migration status and negative self-perceived health.

**Table 3:** Correlates of negative self-perceived health status among migrants without health insurance, Montreal, 2016–2017 (n = 607*).

Variables	OR	95% CI	*P* value
Age	1.05	1.03–1.07	
Marital status			
Married/Common Law	Reference		
Widow/Separated/Divorced	1.02	0.54–1.92	0.945
Single	1.07	0.63–1.82	0.809
Living with children			
No	Reference		
Yes	0.76	0.47–1.23	0.260
Authorized migration status			
Yes	Reference		
No	0.92	0.39–2.16	0.851
Region of birth			
US/Europe	Reference		
Sub-Saharan Africa	2.21	0.89–5.45	0.085
Latin America	1.05	0.48–2.34	0.895
Asia	1.23	0.34–4.45	0.747
Caribbean	0.89	0.36–2.22	0.802
Middle East	2.21	0.96–5.09	0.062
Duration of stay in Quebec			
Less than 5 years	Reference		
More than 5 years	1.26	0.64–2.48	0.501
Education			
University	Reference		
No diploma/Primary or Secondary school	2.49	1.53–4.07	<0.001
Post-secondary non- university	2.41	1.38–4.20	0.002
Type of work contract			
Permanent	Reference		
Temporary	1.06	0.37–3.04	0.909
On call | Self-employed	1.38	0.64–2.98	0.412
Do not work	1.33	0.66–2.69	0.422
Family income meets needs			
Very well/perfectly	Reference		
No/a little	6.22	1.62–23.85	0.008
Fairly	4.70	1.21–18.27	0.025
Insalubrious housing			
No	Reference		
Yes	1.16	0.75–1.80	0.504
Food insecurity			
Never	Reference		
Often | Sometimes	1.17	0.73–1.89	0.506
Discrimination based on origin (Racism)			
No	Reference		
Yes	1.58	1.01–2.48	0.045
Discrimination based on migration status			
No	Reference		
Yes	0.67	0.40–1.14	0.139
Fear/stress in obtaining health care			
No	Reference		
Yes	0.70	0.41–1.21	0.207
Fear/stress of meeting a police agent			
No	Reference		
Yes	0.91	0.56–1.48	0.705
Fear/stress in going to hospital			
Authorized Status	Reference		
Without authorized status–No	2.08	0.80–5.39	0.133
Without authorized status–Yes	1.72	0.73–4.07	0.213
Having someone to talk to			
Yes	Reference		
No	1.34	0.79–2.25	0.277
Having someone to who can be asked for money to			
Yes	Reference		
No	1.60	1.05–2.46	0.030
Do not know	2.90	0.94–8.94	0.064
Kessler Psychological distress			
No	Reference		
Yes	2.17	1.36–3.45	0.001
Unmet health needs			
No	Reference		
Yes	3.45	2.05–5.82	<0.001
Had a health issue in the past 12 months			
No	Reference		
Yes	3.44	1.79–6.61	<0.001
Number of years without medical insurance	0.97	0.91–1.04	1.044

* Includes participants with complete observations for variables of interest. Multivariate analysis adjusted for age

We also assessed correlates for selected variables stratifying the model by gender (Table 4). Variables that were associated with negative self-perceived health differed among men and women. For region of birth, being born in the sub-Saharan African region was significantly associated with higher odds of negative self-perceived health status among males (OR: 4.50, 95% CI:1.11–18.21) while being born in Middle East was associated with the dependent variable among females (OR: 3.93, 95% CI:1.50–10.25). Lower formal education, having a family income that does not meet perfectly or very well needs, insalubrious housing and not knowing someone to ask money to were associated with higher odds of negative self-perceived health among women while not among men. Having had a previous diagnosis of health issues was associated with higher odds of negative self-perceived health status among males than females.

**Table 4:** Correlates of negative self-perceived health status stratified by gender among migrants without health insurance, Montreal, 2016–2017.

Variables	Women (n = 440)		Men (n = 242)	
	OR	95% CI	OR	95% CI
Age	1.04**	1.02–1.05	1.02*	1.00–1.05
Region of birth				
US/Europe	Reference		Reference	
Sub-Saharan Africa	1.96	0.73–5.28	4.50*	1.11–18.21
Latin America	1.20	0.49–2.93	1.12	0.34–3.71
Asia	0.65	0.14–2.85	0.49	0.08–2.97
Caribbean	1.31	0.48–3.54	0.69	0.15–3.09
Middle East	3.93*	1.50–10.25	1.57	0.44–5.57
Education				
University	Reference		Reference	
No diploma/Primary or Secondary school	4.22**	2.39–7.45	1.51	0.74–3.08
Post-secondary non- university	2.03*	1.11–3.71	1.61	0.69–3.75
Family income meets needs				
Very well/perfectly	Reference		Reference	
No/a little	13.50*	1.55–117.61	1.78	0.43–7.34
Fairly	10.08*	1.14–88.71	0.53	0.12–2.39
Food insecurity				
Never	Reference		Reference	
Often | Sometimes	1.40	0.83–2.37	1.40	0.67–2.89
Insalubrious housing				
No	Reference		Reference	
Yes	1.70*	1.04–2.76	1.06	0.56–2.01
Having someone to ask money to				
Yes	Reference		Reference	
No	1.30	0.81–2.08	1.90	0.99–3.65
Do not know	5.32*	1.34–21.04	1.44	0.27–7.67
Previous medical diagnosis (ever)				
No	Reference		Reference	
Yes	2.43**	1.51–3.91	3.62**	1.81–7.24
Number of years without medical insurance	0.99	0.94–1.05	1.07	0.98–1.18

*P value < 0.05

**P value < 0.001

## Discussion

To the best of our knowledge this is the first large empirical study that aimed at examining the social determinants of self-perceived health of immigrants without health insurance residing in Montreal, Quebec. The sample was composed of individuals with a precarious migration status (temporary or unauthorized/undocumented), mostly not working and living with insufficient family income. Self-assessment of general health has a predictive power for morbidity and mortality and has been shown to be a valid indication of general well-being [35]. In this observational study, the proportion of individuals who perceive their own health as negative (44.6%) is substantially higher if compared with other Canadian studies. Nanhou and Bernèche measured the evolution of the health perception of Canadian immigrants and Canadian-born during three periods of time comprised between 2003–2012 [31]. The highest average proportion of people (both men and women) who perceived their health as negative was about 8.5% among recent immigrants (9 years and less), 19.8% among long-term immigrants (10 years or more), and 10.6% among Canadian-born. Using data from Canadian Community Health Surveys (CCHS), researchers found similar estimates: they indicated a decline of self-reported health among immigrants over a 10 years period: from 7.2% (in 2001) to 17.5% (in 2010) who declared poor health.

As expected, in our analysis, the proportion of respondents who perceived their health as negative is higher among those who declared not having an authorized migration status (54%) than those who declared having a temporary status (36%). However, this difference was not statistically significant in the multivariate model. This might be explained by the fact that our sample was generally socio-economically disadvantaged and both those with and without authorized statuses were experiencing precariousness related to social determinants of health such as low family income, lack of social network and racism, all of which can be prominent in determining self-perceived health in these populations. In fact, the proportion of those who declared health issues and unmet health care needs are relatively similar in both categories, and these health variables are strongly correlated with negative self-perceived health. It was already suggested that being uninsured in Canada leads to ‘poorer care and poorer health outcomes’[61]. Another hypothesis could be that the effect of migration status on self-perceived health could be mediated by other determinants of health investigated in our study such as perception of discrimination due to origin (racism), having someone to ask for money to or psychological distress which would in turn have an impact on perceived health. Nanhou and Bernèche estimated that the prevalence of psychological issues was between 3–6% among immigrants and Canadian-born [31]. Based on our analysis, about one quarter (26,3%) of the sample seems to experience mental distress. The differential perception of health observed in our study does not seem to be solely explained by medical issues, but also on social and economic factors, as suggested in our multivariate final model.

Previous studies in Canada suggested that immigrant health is associated with age, gender, ethnic origin, income, access to health care, work stress and health behaviors. In our multivariate analysis, significant correlates of self-perceived health status are: no university diploma, family income that does not meet needs perfectly or very well, not having someone to who can be asked for money, perception of racism, feeling psychological distress, having had a health problem and unmet health care needs in the previous 12 months. Legal migration classifications lead to differential access to resources and opportunities such as, for example, employment and income, decent housing, and to health care and social services or food security [11, 12]. Our study confirms that precarious migration status can represent obstacles to accessing resources (fears and uncertainties related to migration status; inability to obtain a work permit and stable employment; poor working conditions; racism; lack of social support) and reinforce pre-existing structural and institutional barriers (high costs and poor organization of care, language barriers, poor quality of care) [6, 12].

A precarious migration status can lead to poor living conditions that can be deleterious: substandard, unsanitary and overcrowded housing; low income; health-related debts; poor and dangerous working conditions (precarious work, higher risks of work-related accidents) [9]. Furthermore, our sample is mainly composed of recent immigrants, a situation that is known to be associated in Canada with poor social determinants of health [3]. Numerous authors have previously shown that low social position is a risk factor for health [37, 55, 58]. In our study, negative perception of health was higher among respondents who declared insufficient family income. Structural factors such as a low SES are accompanied by a low level of control and little or no rewards that can be a source of chronic stress whose deleterious effect is recognized [62]. As our results also suggest immigrants who do not have social or financial support are more likely to perceive their health as negative. On the contrary, perception of positive health is associated with social, family and friendship networks as it might help to find resources and opportunities [44]. Despite most respondents having an authorized temporary status (ex. long-term visitors, international students), many of these statuses are excluded from public health insurance eligibility, which has consequences on health care access: 68,9% declared unmet health care needs. It is also suggested that the direction and strength of the association between migration status and health outcomes vary across places of birth [37, 39]. Regarding gender, most participants recruited at the clinic were women (72.4%) resulting in a predominantly female sample (63.1%). Such gender differentials in health-seeking behaviors have been described elsewhere [63]. In our final multivariate model stratified by gender, the Middle East and the Sub-Saharan Africa origins are significantly associated with a negative health perception among females and males, respectively. In a study conducted in Belgium, there are higher (perinatal) mortality rates among non-European foreign-born mothers as compared to national population and European-born migrants [27, 39]. Albeit, health outcomes vary between nationalities [30]. It was already suggested that African immigrants encounter considerable challenges and barriers (related to the health system, transportation, employment, language and cultural differences, and lack of social support) in accessing primary health care services in Canada [23]. Migrants from developing countries might have a lower social position in the host country and are potentially exposed to higher vulnerabilities (exploitation, racism) [35]. Racialized minority position and precarious migration status in a country increase the risk of experiencing symptoms of mental illness, and the risk is higher in groups facing deprivation and racism [35, 60]. In our study, those who reported perception of racism were at higher odds of negative perception of health. Racism may have been underestimated due to a sample predominantly composed of recent immigrants whom might have, to date, encountered less exposure to racism and other forms of discriminations but whose deleterious effects is known to increase over time [64,65].

In our study, more than one third of the respondents (34.7%) declared experiencing food insecurity, and this proportion increases to almost half of respondents (44%) without authorized migration status, a situation that is associated with a higher probability of negative self-perception of health. It was already suggested that food insecurity is linked to migration status and poverty, and has short and long-term implications for health, such as, for instance, developing diseases such as tuberculosis, and therefore may impact public health [66]. A substantial proportion of respondents declared experiencing various stressful situations due to the fear of not receiving health care. The proportion of migrants who declared experiencing fear in obtaining health care (70%) is similar to the proportion of respondents who declared unmet health care needs (68.9%). It is possible that stress is associated with not accessing health care, which could influence levels of health perception [67].

For those without authorized status, fear was also associated with trying to access public services and hospitals or encountering immigration officers. Many authors have reported that fear of being denounced or deported, violence in certain domains of social and professional work life, and a difficult family situation (separation, deportation, conflict, death and illness of a parent) are also stressful and have health impacts [8,9]. Obstacles related to the immigration process and delays in the regularization of status can lead to stress and impact on mental health [9].

### Strengths and limitations

The results of the present study present important strengths: first population survey in Canada, large sample, cautious recruitment process. Several limitations may impact the results, however. First, it is difficult to ascertain if the sample is representative of the uninsured population in Montreal as this population is diverse and largely unknown, it is very difficult to identify and there is no census or lists from which to draw a sample. Furthermore, recruitment in a clinic dedicated to uninsured allowed easier identify this population but may have introduced a selection bias towards persons with more health issues. In addition, data were self-reported and may be subject to social desirability bias as the questionnaire was administered by interviewers. However self-rated health status is an important indicator and recognized as a valid and reliable measure associated with functional health, morbidity, mortality, and health service use [57,68]. Because of the cross-sectional design, it is difficult to ascertain whether the health issues declared are consequences of the lack of insurance. However, this association is well known [51] and does not need to be demonstrated. It was not possible to include all variables of interest in our gender-stratified multivariate analysis as their distribution varied according to gender, not enabling us to control for confounding. As a result, some variables of conceptual importance might have been missed in the analysis. However, the most important ones were included (socio-economic, demographic, psychosocial, and health characteristics). The lack of statistical power might have had an impact on our ability to detect association of low size effect in the regression logistic model among men, based on simulations performed using Cohen’s d and GPower [69]. Finally, out of fear of repercussions, some participants may have reported having a temporary status when they in fact were without authorized status. Consequently, our estimates might be conservative: the situations of those with and without authorized status are likely more similar in our study than they are in reality, and we could have found more significant results without this potential bias.

## Conclusions

This cross-sectional study suggests that the health perception of uninsured migrants with precarious status living in Montreal is poor, particularly as compared to results of previous studies on the health perception of Canadian immigrants and Canadian-born. Our results do not show a significant difference in terms of negative self-perceived health between subcategories of migrants with precarious status; this could mean that both the socioeconomic precariousness (associated with authorized and unauthorized statuses), and being uninsured might be responsible for poor health. This suggests that the Quebec government's immigration plan, which they articulate as the welcoming, integration and participation of permanent immigrants in their new society for the prosperity of the province [70], targets only some while others seem excluded from many opportunities and resources. The circumstances that make migration precarious [15] or irregular [29] are structural (informal economy, lack of legal migration avenues or overly restrictive immigration procedures and laws) confirming that any framework related to migration and health should consider power relationships that contribute to shaping social position [22].

Regarding gender, our sample was mainly made up of women (63.6%). Our results suggest two kind of interactions: between gender and region of origin on the one hand, and between gender and SES on the other hand. Being women from Middle East origin and men born in Sub-Saharan African were both correlated with higher odds of negative self-perceived health. Furthermore, lower formal education and lower family income are associated with negative self-perceived health among women only.

Our study also indicates a high rate of unmet health care needs among migrants with precarious status, a situation that is correlated with poor health. This obvious inequitable structural treatment of human beings, such as the exclusion of some migrants from public health coverage, is in complete contradiction with international, national and even provincial human rights principles, social justice and human dignity [71–73].

The present study also suggests that a high proportion of migrants experienced racism (51.9%), which significantly increases the likelihood to perceive one’s health as negative. The context of immigration in Canada is challenging for many, especially for racialized minorities, who experience various challenges and difficulties that can have deleterious consequences and negatively impact well-being [28]. Canada’s system of increasingly complex migration statuses, many of which are precarious, has implications not only for the health of the migrant population through structural and psychosocial mechanisms but also for public health. Social policies that secure access to resources, health care and social services for all immigrants, with or without authorized status, are an absolute requirement not only for migrant well-being but also for overall public health [66].

## Supporting information

S1 Fig Achieved power for α = 0.05, a sample size of 607 participants and a small effect size. (DOCX)

S2 Fig Achieved power for α = 0.05, a sample size of 607 participants and a medium effect size. (DOCX)

S3 Fig Achieved power for α = 0.05, a sample size of 607 participants and a large effect size. (DOCX)

S4 Fig Achieved power for α = 0.05, a sample size of 440 participants and a small effect size. (DOCX)

S5 Fig Achieved power for α = 0.05, a sample size of 440 participants and a medium effect size. (DOCX)

S6 Fig Achieved power for α = 0.05, a sample size of 440 participants and a large effect size. (DOCX)

S7 Fig Achieved power for α = 0.05, a sample size of 242 participants and a small effect size. (DOCX)

S8 Fig Achieved power for α = 0.05, a sample size of 242 participants and a medium effect size. (DOCX)

S9 Fig Achieved power for α = 0.05, a sample size of 242 participants and a large effect size. (DOCX)
