# Supplementary material for: The negative self-perceived health of migrants with precarious status in Montreal, Canada: A cross-sectional study
Source: PLoS One. 2020 Apr 9;15(4):e0231327. doi: 10.1371/journal.pone.0231327 (PMC7145148; doi:10.1371/journal.pone.0231327)
Supplement: S1 Fig — (DOCX) [file pone.0231327.s001.docx]

1. **Statistical Power Analyses for logistic regression from table 3**

- **Achieved power for α = 0.05, a sample size of 607 participants and a small effect size.**

(Small effect size: Cohen’s d = 0.2 🡪 Odds ratio = 1.44)


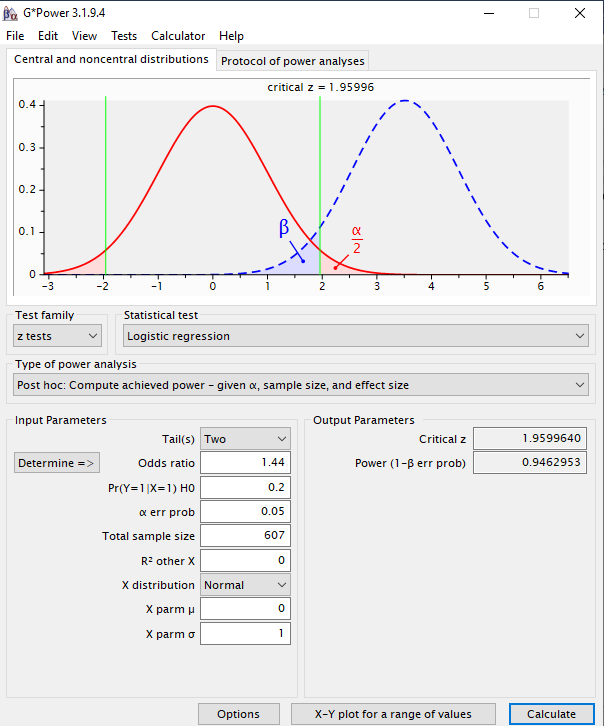


Figure 1. Achieved power for α = 0.05, a sample size of 607 participants and a small effect size.

***Comment:*** For a small effect size, α = 0.05, a sample size of 607 participants, the achieved power was 94.6% for identifying predictors.

1. **Statistical Power Analyses for logistic regression from table 4 : Sex = Men**

- **Achieved power for α = 0.05, a sample size of 242 participants and a small effect size.**

(Small effect size: Cohen’s d = 0.2 🡪 Odds ratio = 1.44)

- **Achieved power for α = 0.05, a sample size of 242 participants and a medium effect size.**

(Medium effect size: Cohen’s d = 0.5 🡪 Odds ratio = 2.48)

- **Achieved power for α = 0.05, a sample size of 242 participants and a large effect size.**

(Large effect size: Cohen’s d = 0.8 🡪 Odds ratio = 4.27)

**REFERENCES**

1. G*Power Manual: Statistical Power Analyses for Windows and Mac [Internet]. 2017. Available from: http://www.gpower.hhu.de/fileadmin/redaktion/Fakultaeten/Mathematisch-Naturwissenschaftliche_Fakultaet/Psychologie/AAP/gpower/GPowerManual.pdf

2. Faul F, Erdfelder E, Buchner A, Lang A-G. Statistical power analyses using G*Power 3.1: Tests for correlation and regression analyses. Behavior Research Methods. 2009 Nov 1;41(4):1149–60.

3. Borenstein M, editor. Introduction to meta-analysis. Chichester, U.K: John Wiley & Sons; 2009. 421 p.
