# Supplementary material for: The negative self-perceived health of migrants with precarious status in Montreal, Canada: A cross-sectional study
Source: PLoS One. 2020 Apr 9;15(4):e0231327. doi: 10.1371/journal.pone.0231327 (PMC7145148; doi:10.1371/journal.pone.0231327)
Supplement: S4 Fig — (DOCX) [file pone.0231327.s004.docx]

1. **Statistical Power Analyses for logistic regression from table 4 : Sex = Women**

- **Achieved power for α = 0.05, a sample size of 440 participants and a small effect size.**

(Small effect size: Cohen’s d = 0.2 🡪 Odds ratio = 1.44)


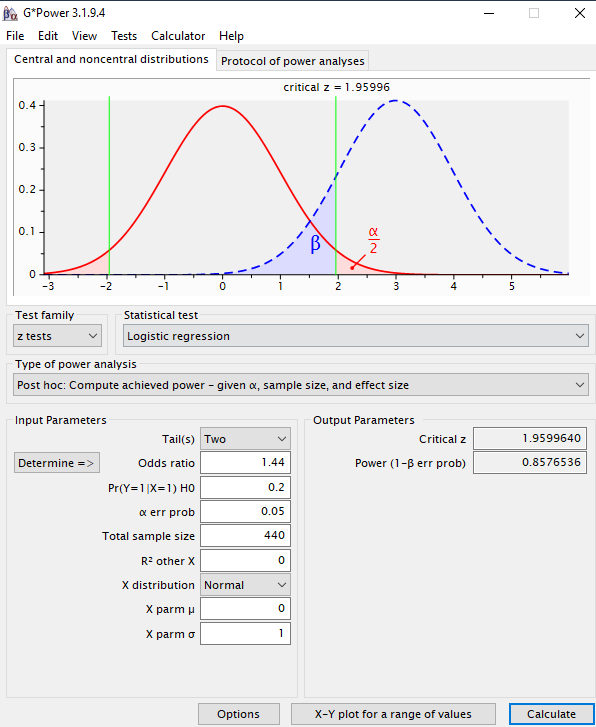


Figure 4. Achieved power for α = 0.05, a sample size of 440 participants and a small effect size.

***Comment:*** For a small effect size, α = 0.05, a sample size of 440 participants, the achieved power was 85.8% for identifying predictors.
