# Supplementary material for: The negative self-perceived health of migrants with precarious status in Montreal, Canada: A cross-sectional study
Source: PLoS One. 2020 Apr 9;15(4):e0231327. doi: 10.1371/journal.pone.0231327 (PMC7145148; doi:10.1371/journal.pone.0231327)
Supplement: S6 Fig — (DOCX) [file pone.0231327.s006.docx]

- **Achieved power for α = 0.05, a sample size of 440 participants and a large effect size.**

(Large effect size: Cohen’s d = 0.8 🡪 Odds ratio = 4.27)


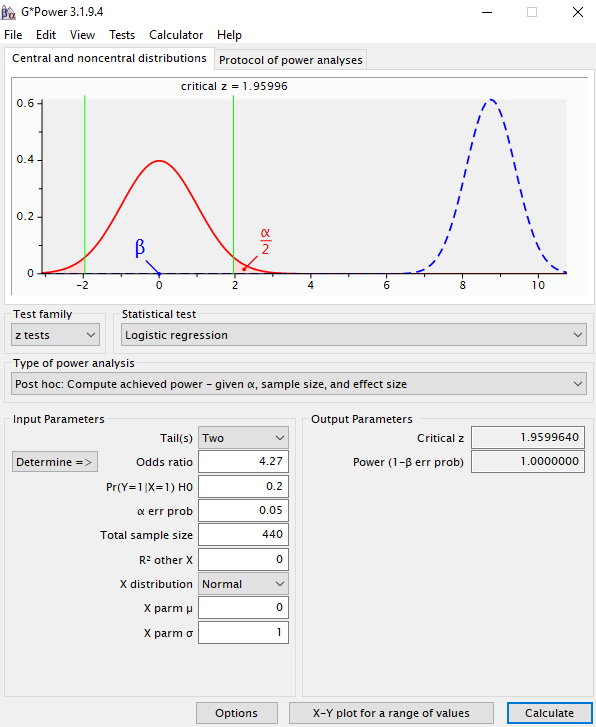


Figure 6. Achieved power for α = 0.05, a sample size of 440 participants and a large effect size.

***Comment:*** For a large effect size, α = 0.05, a sample size of 440 participants, the achieved power was 100% for identifying predictors.
