# Supplementary material for: The negative self-perceived health of migrants with precarious status in Montreal, Canada: A cross-sectional study
Source: PLoS One. 2020 Apr 9;15(4):e0231327. doi: 10.1371/journal.pone.0231327 (PMC7145148; doi:10.1371/journal.pone.0231327)
Supplement: S7 Fig — (DOCX) [file pone.0231327.s007.docx]

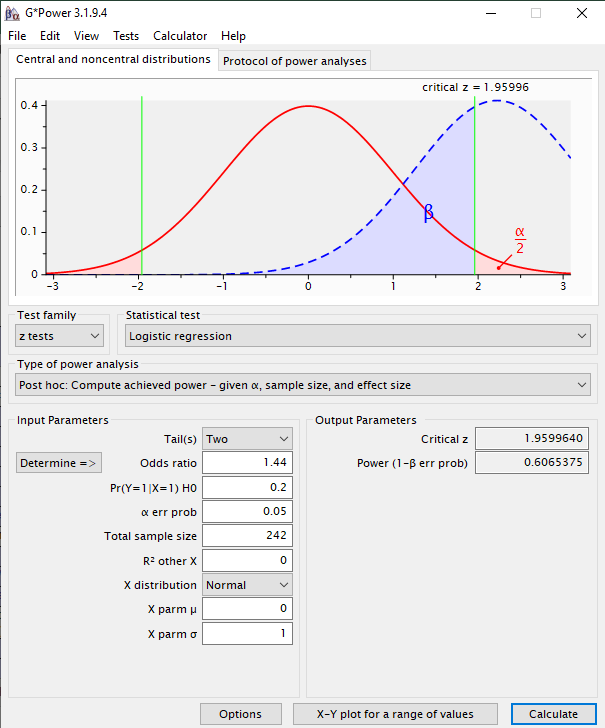


Figure 7. Achieved power for α = 0.05, a sample size of 242 participants and a small effect size.

***Comment:*** For a small effect size, α = 0.05, a sample size of 242 participants, the achieved power was 60.6% for identifying predictors.
